# The Proposed Royal Society of Medicine

**Published:** 1905-03-11

**Authors:** 


### THE PROPOSED ROYAL SOCIETY OF MEDICINE.

The genius of the Northern races has been adverse to centralisation from time immemorial, and the English nation has never proved itself essentially different from the parent stock. It has always seemed better to us to be governed locally than to submit to a bureaucracy, and what is true in the world of politics is equally true in medicine. Hence it is that we still retain the many portal system of qualification which is at once the wonder and the stumbling-block of our French and Italian confrères, who never understand how so many medical men practise in England and are without the title of doctor. Amongst the medical societies of London the tendency has always been in the direction of disintegration rather than of concentration. The Medical Society was by no means the only medical society in London during the later part of the eighteenth century, but it is almost the only one which has survived to the present day with its faculties and finances unimpaired. The Royal Medical and Chirurgical Society arose from it as an offshoot in 1805 in consequence of a disagreement on matters of internal economy, and from the Medical and Chirurgical Society came the Pathological Society in 1846, the Obstetrical Society in 1858, and the Clinical Society in 1867. The Medical Society must therefore be looked upon as the parent, though the Medical and Chirurgical has been the more prolific. The process of decentralisation has gone on uninterruptedly for more than one hundred years in spite of attempts which have been made at various times to arrest it. The last serious attempt was ended with the death of Sir Andrew Clark, but the recent address given by Sir R. Douglas Powell, the president of the Royal Medical and Chirurgical Society, draws attention again to the desirability of amalgamating at least the chief medical societies in London and perhaps getting them incorporated upon somewhat similar lines to the Royal Academy of Medicine in Ireland or the French Académie at Paris. There is at present a great waste of energy, material, money, and time. The same subject may be discussed in the course of a single session at several different societies. The reports of the discussions and the conclusions obtained are widely separated, are published late and are often buried in transactions which have only a limited circulation, so that it is difficult to ascertain the opinions of the individual speakers, in spite of the condensed reports which are inserted in the medical periodicals. Money spent in the hiring of the various meeting places and that spent in the publication of separate volumes of Transactions might be spent to much greater advantage if the different societies were amalgamated, whilst the cost to the members would be materially diminished, for, instead of subscribing to many societies a single subscription would suffice. We welcome, therefore, the proposal, which was carried unanimously at the annual meeting of the Royal Medical and Chirurgical Society, that "the Council of the Society be requested with as little delay as possible to invite the leading medical societies of London to arrange for a joint meeting for the purpose of considering the advisability of amalgamating and to take the necessary preliminary steps for the purpose." Many difficulties must necessarily be encountered. There is no doubt that it will be impossible to federate all the societies, for there are many with a local habitation, recruited almost entirely from local sources, who would find it most inconvenient to adopt a distant place of meeting. The financial questions involved will have to be considered most carefully and must be treated in a liberal spirit on all sides. The adequate representation on a central council may prove a matter of extreme difficulty, whilst the bylaws and the method of publishing will require careful thought and considerable technical knowledge and business capacity. Yet all these difficulties may be overcome by tact, care, and the deliberation of a small committee if the proper members be selected. The present time would seem to be appropriate for the plan to be entertained, and, if possible, carried to a successful issue, because for a time the talk is all of union and fusion. The Royal Medical and Chirurgical Society of London is marked out with equal clearness to father the scheme. It is financially stable, it is now celebrating its centenary, and it alone has adequate premises wherein the different branches might be housed.

